# Numerical Analysis and Design of an Ultra-Thin Flexible Transparent Metasurface for Broadband Radar-Infrared Compatible Stealth

**DOI:** 10.3390/mi17030277

**Published:** 2026-02-24

**Authors:** Liang Xu, Yijia Li, Xingyuan Wang, Jingxuan Sun, Zhixun Yang

**Affiliations:** 1 College of Mechanical and Electrical Engineering, Harbin Engineering University, Harbin 150001, China; 2 College of Information and Communication Engineering, Harbin Engineering University, Harbin 150001, China

**Keywords:** metasurface, radar-infrared compatible stealth, microwave absorption, infrared shielding, visible-light transparency

## Abstract

In response to the significant challenges posed by the rapid progress of multi-spectral detection technologies to traditional stealth techniques, this paper presents a flexible transparent metasurface structure that is compatible with radar and infrared stealth. It consists of multi-layer functional patterned indium tin oxide (ITO) films and a flexible polydimethylsiloxane (PDMS) substrate. The metasurface uses a high-duty-cycle multi-scale circular ring to achieve a microwave absorption bandwidth of 30 GHz and low infrared emissivity of 0.33 in an optimized ultra-thin 2.65 mm thickness system. The simulation results show that the metasurface achieves absorption exceeding 90% in the frequency range of 10.8–40.8 GHz, which covers common radar bands like X, Ku, K, and Ka. Furthermore, the structure exhibits polarization insensitivity and sustains stable absorption in a wide range of 60 degrees of transverse magnetic (TM) fields. Meanwhile, it decreases the radar cross-section (RCS) by more than 10 dB over a wide angular range even when bent. This study presents a feasible metasurface with ultra-thin, flexible, transparent, and multi-spectral compatibility for the next generation of stealth systems.

## 1. Introduction

The rapid development of multi-spectral composite detection technologies such as radar, infrared and visible light has advanced modern detection systems from single-band sensing to integrated multi-source fusion-based detection. These systems offer an improved resolution, increased target accuracy and weatherproof operation. One of the most popular composite detection techniques is the combination of infrared and radar tracking [1,2,3]. This trend increases the exposure risk of targets that rely on traditional single-band stealth techniques, and thus it creates an urgent need for multi-band compatible stealth [4,5,6,7].

In recent years, metasurfaces, artificially constructed periodic subwavelength structures, have emerged as a novel technical approach for achieving multi-band compatible stealth due to their powerful control of the reflection, transmission, absorption, and phase of electromagnetic waves [8,9,10,11,12,13,14]. Phase-modulation-based and absorption-based radar stealth strategies are the two main techniques based on metasurfaces [15,16,17,18]. Phase-modulating metasurfaces control the direction and intensity of radar scattering by precisely manipulating the reflected phase to cause destructive interference or reroute scattered waves [19,20,21,22,23]. This makes the phase modulation metasurface more sensitive to the accuracy of the unit, the angles of incidence and the polarization states, reducing its bandwidth and angle performance, while preventing direct compatibility with infrared radiation management. In contrast, absorptive metasurfaces, by converting incident radar wave energy into heat via electromagnetic resonance and material loss, successfully reduce the intensity of radar echo [24,25,26,27,28,29]. They demonstrate more stable radar suppression in complex electromagnetic environments by directly attenuating incident energy and provide a structural platform for integrating infrared control functions, making them a popular approach for broadband radar stealth and multi-spectral compatibility.

The two most common configurations of absorption-based radar-infrared multi-spectral compatible metasurfaces are sandwich-type and frequency-selective surfaces (FSSs). Sandwich-type integrated metasurfaces achieve absorption or modulation by a single functional layer, and they offer a compact structure that is more conducive to optical transparency and flexible application [21,30]. For example, Wang et al. [31] designed an ITO-dielectric-ITO metasurface achieving a −10 dB radar cross-section (RCS) reduction across 7.4–13.4 GHz, with an infrared emissivity of approximately 0.26 and an overall thickness of 3.35 mm. However, due to limited resonant modes, the absorption bandwidth of integrated single-layer metasurfaces is constrained, covering only the X-band. In contrast, FSSs employ multi-functional layers to achieve infrared stealth and microwave absorption, respectively [32]. By stacking these functional layers, multiple resonant modes can be formed to broaden the radar absorption bandwidth [27,33,34,35,36]. Duan et al. [29] proposed a flexible layered structure based on an aluminum foil patch metasurface with a carbonyl iron/polyurethane coating. This structure achieves over 90% absorption in frequency bands spanning 3.7–5.1 GHz and 12.8–18 GHz, with an infrared emissivity of 0.281 and a total thickness of 2.2 mm. However, optical transparency is sacrificed due to the use of high-dielectric-loss media. Zheng et al. [25] proposed a transparent rigid absorber with absorption frequency band range of 2.2–18 GHz based on multi-layer ITO conductive films. It has a visible light transmittance of about 40% and a final thickness of 12 mm. Although it offers an excellent bandwidth, the pursuit of a high bandwidth comes at the cost of high thickness, which also compromises flexibility and conformal integration on curved surfaces. Therefore, new metasurfaces should be explored to solve the problem of compatibility of radar and infrared stealth mechanisms, allowing the design of high-performance stealth devices with wideband compatibility, curved surface compliance and lightweight ultra-thinness.

This paper proposes a radar-infrared multi-spectral compatible flexible metasurface. It is based on multi-layer patterned indium tin oxide (ITO) films, working in concert with underlying flexible polydimethylsiloxane (PDMS) substrate. The infrared stealth layer is made of high-duty-cycle annular perforated ITO patches. It is effectively integrated with the underlying ring-cross-patterned microwave absorption layer through synergistic optimization of multi-scale geometric parameters and impedance distribution in an ultra-thin system. The metasurface provides a broadband radar wave absorption, while maintaining high optical transmittance and excellent flexibility. Meanwhile, the electromagnetic loss mechanisms, polarization and incidence angle stability, and performance under bending conditions are also presented. This provides a viable solution with ultra-thin, flexible, and transparent characteristics for the next-generation multi-spectral stealth systems like optical windows and complex curved surfaces.

## 2. Absorber Structure and Simulation Setup

### 2.1. Absorber Structure

**Figure 1:**
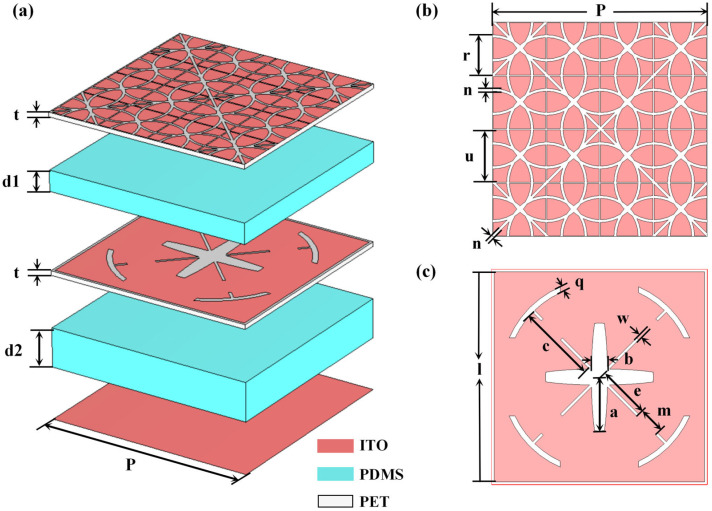
Schematic diagram absorber structure design: (**a**) unit cell schematic diagram; (**b**) top view of infrared stealth layer structure; (**c**) top view of radar absorption layer structure.

Figure 1a shows the structure of the proposed metasurface absorber. The unit cell consists of an infrared stealth layer, a radar absorption layer, and a total reflection layer, separated by a PDMS dielectric layer, sequenced from the top to the bottom. All materials used in the structure are optically transparent to meet optical transparency requirements. The infrared stealth layer, radar absorption layer, and total reflection layer utilize ITO films with different sheet resistances. The ITO films of the infrared stealth layer and radar absorption layer are patterned into the designed structure. Figure 1b and Figure 1c show the unit geometries of the infrared stealth layer and radar absorption layer, respectively. The substrate for ITO patterned films is polyethylene terephthalate (PET) with a thickness of *t* and a dielectric constant of 3 + j0.06. The top and bottom substrate layers employ flexible PDMS with high optical transmittance (>95%), a dielectric constant of 2.7 + j0.001, and thicknesses *d*1 and *d*2, respectively. All these layers can be fabricated using large-scale manufacturing techniques. For example, the ITO patterns of the infrared stealth layer, radar absorption layer and total reflection layer can be fabricated by spraying ITO on PET using a shield mask with the same design as the corresponding layers, while PDMS can be produced by casting. The final metasurface can be assembled by successive bonding or adhesion of these layers. This configuration ensures high optical transmittance while providing structural support and excellent mechanical flexibility. The parameters of the metasurface absorber are shown in Table 1.

### 2.2. Simulation Settings

The electromagnetic simulation software CST Studio Suite 2022 was used to test the electromagnetic performance of the metasurface. Modeling and numerical simulation were performed using the parameters of Table 1. During simulation, a frequency-domain solver was selected to balance computational accuracy and efficiency. Boundary conditions were set as a unit cell periodic structure to simulate an infinitely periodic metasurface array. Floquet ports were used to excite x-polarized and y-polarized electromagnetic waves incident along the +z direction, with simulation analysis conducted in the frequency range of 5–45 GHz. For the transverse electric (TE) oblique incidence mode, the electric field direction of the incident electromagnetic wave aligned along the *X*-axis, while the magnetic field direction aligned along the *Y*-axis. For the transverse magnetic (TM) oblique incidence mode, the electric field direction aligned along the *Y*-axis, while the magnetic field direction aligned along the *X*-axis.

## 3. Results and Discussion

### 3.1. Design of the Infrared Stealth Layer

For radar-infrared multi-spectral stealth metasurfaces, the primary design objective of the infrared stealth layer is to effectively reduce the thermal radiation signature in the mid- and high-frequency infrared bands while maintaining the high visibility of the light without compromising the radar absorption performance of the background. Therefore, the design of this layer requires a comprehensive trade-off between infrared emissivity, microwave transmission characteristics, and optical transparency. From the point of view of material selection, ITO films are commonly used as transparent conductive materials in infrared stealth structures because of its high visibility and reflective properties in the infrared band. The dielectric constant of ITO in the infrared band can be described by the Drude model [37]:

(1) $$\varepsilon (\omega )=\varepsilon_{b}-\frac{\omega_{p}^{2}}{\omega (\omega +i\omega_{c})}$$

where *ε*_b_ represents the high-frequency dielectric constant, and ω_p_ and ω_c_ denote the plasma frequency and collision frequency, respectively. According to the Drude model, the real part of the dielectric constant of ITO becomes negative. This confers it metallic-like reflective properties, effectively suppressing thermal radiation and reducing infrared emissivity [38]. This makes ITO an ideal material choice for achieving infrared stealth functionality without significantly affecting visible light transmittance. However, continuous low-resistance ITO films exhibit high equivalent conductivity in the microwave band, leading to pronounced impedance mismatch with free space and consequently strong microwave reflection. This impedes effective coupling of electromagnetic waves to the underlying radar absorption layer, conflicting with overall radar stealth functionality. Therefore, while achieving low infrared emissivity, the infrared stealth layer must also possess an excellent microwave transmission capability to ensure that the subsequent absorber layer can function optimally [12].

**Figure 2:**
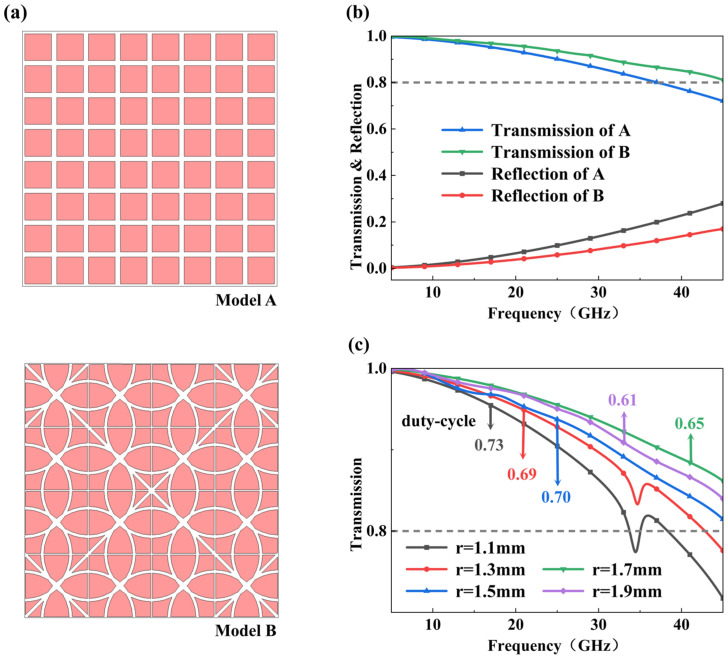
Schematic patch structure and corresponding transmittance and reflectance properties: (**a**) conventional single-size rectangular patch structure (top) and annular hollow multi-scale patch structure (bottom); (**b**) transmittance and reflectance of the two infrared stealth layer structures; (**c**) transmittance with varying *r*.

This paper introduces geometrically perforated structures into the ITO film with a sheet resistance of 6 Ω/sq as the infrared stealth layer to modulate its equivalent electromagnetic response in the microwave frequency band. The electromagnetic response of periodic conductive patches can be approximated by the standing wave theory in the microwave frequency band. For periodic patch structures, the fundamental mode’s resonance frequency is primarily determined by the patch’s equivalent resonant length and equivalent dielectric constant, which is expressed as [38,39]

(2) $$f=\frac{c}{2nw}$$

where c represents the speed of light in a vacuum, w denotes the equivalent width of the ITO patch, and n is the refractive index of the substrate. This relationship suggests that, in the case of fixed material parameters, reducing the equivalent size of the patch or introducing multi-part structures shifts the resonance frequency towards higher frequencies. Accordingly, a novel periodic annular perforated multi-scale patch structure (Figure 2a, bottom) is proposed as a topological evolution of the traditional single-scale rectangular ITO patch (Figure 2a, top). Due to its unique geometric scale, the conventional rectangular patch inevitably excites a strong parasitic resonance at a specific frequency, as shown in Figure S3. As the frequency approaches resonance, the layer suffers impedance mismatch and turns into a reflective surface, blocking the absorption of radar. To mitigate this effect, the conductive region was first segmented into rectangular sub-patches with different sizes, which partially disperses the resonant response and reduces the peak amplitude. Building upon these findings, geometry was further evolved into a multi-scale annular perforated topology. The concentric rings of the multi-scale annular divide the patch into multiple sub-units while preserving fourfold rotational symmetry. The intersections of concentric rings create sub-units that vary in size, which further stabilizes the equivalent surface impedance across a broad spectrum while maintaining the high conductive filling ratio required for infrared stealth. As shown in Figure 2b, compared to the conventional rectangular patch, the designed multi-scale patch exhibits a superior microwave transmission performance and lower reflection levels with the same duty cycle of 70%. This trend becomes more pronounced as the frequency increases from 5 to 45 GHz. This validates the effectiveness of this patterning strategy in balancing infrared stealth and microwave transmission capabilities.

The influence of the radius *r* of the novel periodic annular perforated multi-scale patch structure was further analyzed, focusing on the microwave transmission performance of the infrared stealth layer. Figure 2c shows the microwave transmittance spectra over 5–45 GHz with unit cell *r* values ranging from 1.1 mm to 1.9 mm in steps of 0.2 mm (Figure S1 shows the corresponding structure). As *r* increases, the average microwave transmittance exhibits a clear enhancement, typically at higher frequencies. When *r* ≥ 1.5 mm, broadband microwave transmittance exceeding 80% is achieved across the entire frequency range. This improvement is primarily attributed to the reduction in the effective conductive area of the ITO film with increasing *r*, which weakens microwave shielding and facilitates electromagnetic energy coupling into the underlying radar absorption layer. However, excessively large annular openings (*r* > 1.5 mm) markedly reduce the ITO duty cycle, leading to increased infrared emissivity. Considering microwave transparency and infrared stealth performance, a radius of *r* = 1.5 mm is selected as the final geometric parameter.

### 3.2. Design of the Radar Absorption Layer

The radar absorption layer is designed to efficiently absorb microwaves transmitted from the infrared stealth layer, to significantly suppress the target’s radar scattering signature. This objective must be achieved across the broadest possible frequency range while satisfying multiple constraints: ultra-thin, optical transparency, and compatibility with a flexible substrate. From the perspective of absorption mechanisms, efficient electromagnetic wave absorption typically relies on two key factors: First, achieving good impedance matching between the overall structure and free space, which facilitates the electromagnetic waves to encounter the structure. Second, the structure has a sufficiently strong energy loss inside to convert the electromagnetic energy into heat and dissipate it. Therefore, a design strategy of a synergistic effect between electromagnetic resonance and ohmic losses within the microwave band is introduced. The unit cell of the radar absorption layer, as shown in Figure 1c, is designed by carefully rationalizing its surface resistance and geometric configuration.

From an overall structure perspective, the upper infrared stealth layer functions as a highly efficient microwave transmission layer, maintaining a consistently high transmission rate of more than 80% in the range of 5–45 GHz. The infrared stealth layer, radar absorption layer and the bottom continuous ITO reflector layer separated by the PDMS dielectric layer together constitute a typical absorber system of the transmission–dissipation–reflection type. Firstly, the effects of the thicknesses of the two PDMS dielectric layers, *d*1 and *d*2, on the absorption and impedance performance were analyzed. Absorption *A*(*ω*) is employed to characterize the shield performance of the metasurface against electromagnetic waves in the radar frequency range, described by Equation (3):

(3) $$A(\omega )=1-R(\omega )-T(\omega )=1-|S_{11}{|}^{2}-|S_{21}{|}^{2}$$

where *R*(*ω*) and *T*(*ω*) represent the reflectivity and transmittance of the structure, respectively. Since the continuous ITO reflective layer with a sheet resistance of 6 Ω/sq behaves as an ideal conductor in the microwave frequency band, the transmittance *T*(*ω*) can be considered to be zero. Therefore, the absorption coefficient can be approximated as

(4) $$A(\omega )\approx 1-|S_{11}{|}^{2}$$

**Figure 3:**
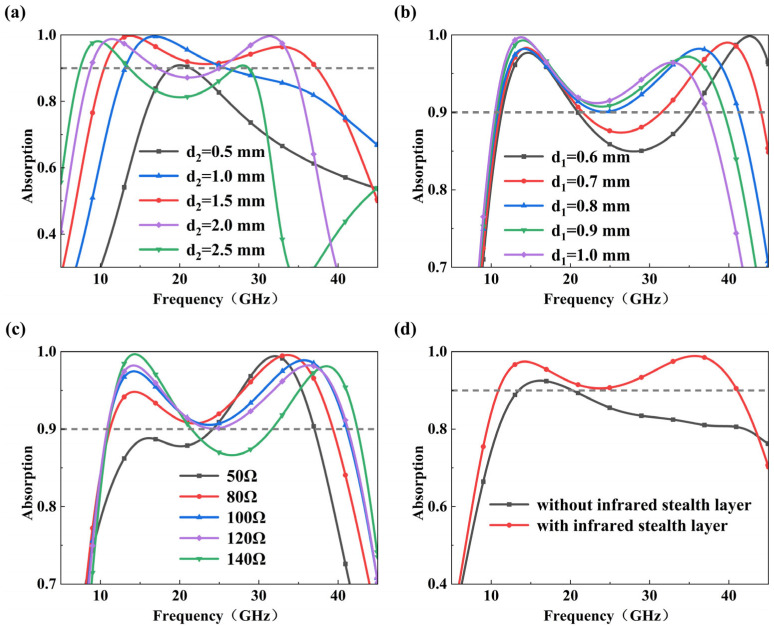
Influence of optimization parameters on absorption performance: (**a**) with different *d*2 at a fixed *d*1 = 1.0 mm and a radar absorber sheet resistance *Rs* = 120 Ω/sq; (**b**) with different *d*1 at a fixed *d*2 = 1.5 mm and *Rs* = 120 Ω/sq; (**c**) with different *Rs* at a fixed *d*1 = 0.8 mm and *d*2 = 1.5 mm; (**d**) compares absorption spectra with and without infrared stealth layer at a fixed *d*1 = 1.0 mm, *d*2 = 1.5 mm and *Rs* = 100 Ω/sq.

Figure 3a shows the absorption performance with different thicknesses *d*2, at a fixed *d*1 = 1.0 mm and a radar absorber layer resistance of 120 Ω/sq. As *d*2 gradually increases to 1.5 mm, the absorption bandwidth is significantly extended, and the resonance frequency shifts toward lower frequencies due to the increased equivalent inductance of the structure. Further increasing *d*2 leads to impedance mismatch with free space, resulting in a narrowed effective bandwidth. Thus, at *d*2 = 1.5 mm, an optimal balance between low-frequency absorption enhancement and broadband impedance matching is achieved. Accordingly, the absorption performance was further investigated for different thicknesses *d*1, at a fixed *d*2 = 1.5 mm and a radar absorber layer resistance of 120 Ω/sq. As shown in Figure 3b, the high-frequency absorption peak gradually approaches the dominant low-frequency resonance as *d*1 increases, enhancing mode coupling and broadening the absorption band. When *d*1 = 0.8 mm, the two resonant modes merge to form a continuous and flat broadband absorption profile. Further increases in *d*1 cause excessive redshift of the high-frequency mode, weakening resonance coupling and reducing bandwidth. Therefore, *d*1 = 0.8 mm is selected as the optimized parameter.

The sheet resistance of the patterned ITO film in the radar absorption layer not only influences the ohmic losses but also the level of impedance matching. Thus, the absorption performance was analyzed for different sheet resistances ranging from 50 to 140 Ω/sq at a fixed *d*1 and *d*2 of 0.8 mm and 1.5 mm, respectively. As shown in Figure 3c, increasing resistance from 50 to 140 Ω/sq generally enhances the absorption due to increased ohmic losses associated with surface current dissipation. The optimal performance is obtained at 100 Ω/sq, where absorption exceeds 90% over a wide frequency range. Further increases in sheet resistance deteriorate impedance matching, particularly in the mid-frequency region, resulting in reduced absorption efficiency and a narrowed effective bandwidth. Therefore, 100 Ω/sq was selected as the optimal sheet resistance, as it achieves an optimal compromise between surface current dissipation and free-space impedance matching. Given the effect of sheet resistance on the absorption performance and their sensitivity to the variations in the spraying process, strict control of spraying parameters is necessary.

The synergies between the infrared stealth layer and radar absorption layer were further analyzed with optimized parameters of *r*, *d*1, *d*2, and radar absorption layer sheet resistance at 1.5 mm, 0.8 mm, 1.5 mm and 100 Ω/sq (unless otherwise specified, this parameter is used in the following discussion). For the radar absorption layer, a hybrid-width modified asterisk enclosed by an outer ring was designed (Figure 1c), where the thick cross defines the basic band, while the thin diagonal arms and outer ring further enhance the low-frequency resonance. Performance gains after adding them consecutively are shown in Figure S4. Figure 3d compares the radar absorption performance of the structure with and without the infrared stealth layer. The standalone radar layer exhibits limited bandwidth dominated by the low-frequency mode. Integrating the infrared layer introduces a secondary high-frequency resonance, and constructive coupling between the top annular grid and the bottom hybrid resonator merges these modes, effectively extending the absorption bandwidth.

### 3.3. Infrared and Radar Stealth Performance

The electromagnetic performance of the proposed metasurface is further comprehensively evaluated based on the optimized parameter. In the infrared spectrum, the overall structure’s equivalent infrared emissivity can be estimated as [40]

(5) $$\varepsilon_{absorber}=\varepsilon_{ITO}S_{ITO}+\varepsilon_{PET}(1-S_{ITO})$$

where *ε*_ITO_ and *ε*_PET_ represent the infrared emissivity of the ITO film and PET substrate, respectively, while *S*_ITO_ denotes the area fraction of ITO. For an ITO film with a sheet resistance of 6 Ω/sq, its infrared emissivity is approximately 0.1, while that of the PET substrate is about 0.877. The calculated equivalent infrared emissivity of this optimized infrared stealth layer is approximately 0.33, meeting the normal requirements for infrared invisibility.

**Figure 4:**
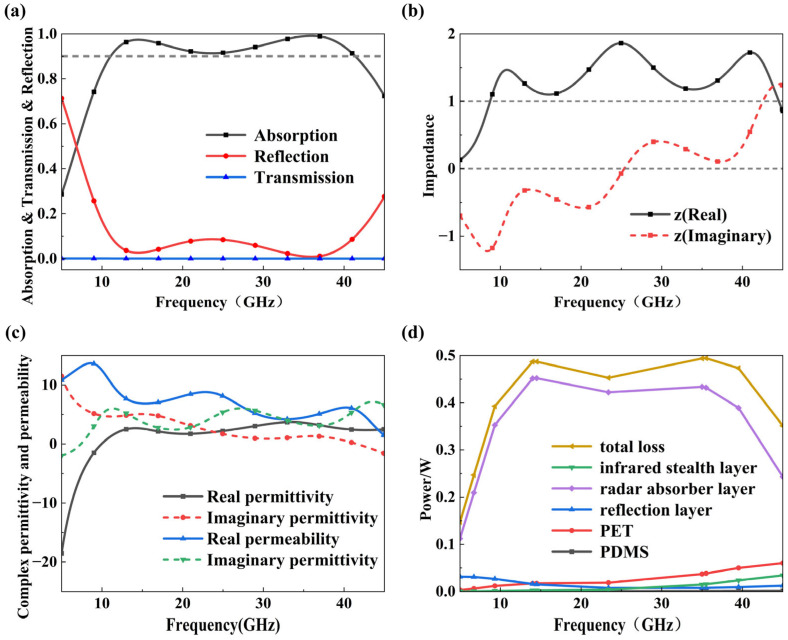
The characteristics of the absorber: (**a**) absorption, transmission, and reflection; (**b**) normalized surface impedance; (**c**) complex permittivity and permeability; (**d**) the energy loss for each layer.

Figure 4a shows the radar absorption, transmission, and reflection of the optimized absorber. It indicates that under perpendicular incidence, the metasurface maintains an absorption rate exceeding 90% across a broad frequency range of 10.8 to 40.8 GHz, with a corresponding absolute bandwidth of approximately 30 GHz and with a relative bandwidth reaching 116.3%. This covers several typical operating radars, including X, Ku, K, and Ka, demonstrating that the designed radar absorber achieves stable and highly efficient broadband absorption under ultra-thin conditions.

An inverse analysis of equivalent electromagnetic parameters was performed from an equivalent medium perspective to elucidate the broadband absorptive mechanism of the designed radar absorber. Under subwavelength conditions, its overall electromagnetic response can be approximated as a homogeneous medium layer, with its equivalent impedance and equivalent dielectric/magnetic parameters obtainable via *S*-parameter inversion. To obtain non-zero *S*_21_ values, rectangular apertures electrically much smaller than the wavelength with a length of 0.2 mm were introduced at the four corners of the total reflection layer. The introduced apertures only serve numerical parameter extraction, without altering the resonance mechanism or absorption characteristics. The equivalent impedance *Z*, dielectric constant *ε*_eff_, and magnetic permeability *μ*_eff_ of the absorber were calculated using Equations (6)–(9):

(6) $$n=\frac{1}{k_{0}d}\arccos\left[\frac{1}{2S_{11}}(1-S_{11}^{2}+S_{21}^{2})\right]$$

(7) $$Z_{eff}=\sqrt{\frac{(1+S_{11}{)}^{2}-S_{21}^{2}}{(1-S_{11}{)}^{2}-S_{21}^{2}}}$$

(8) $$\varepsilon_{eff}=\frac{n}{Z_{eff}}$$

(9) $$\mu_{eff}=nZ_{eff}$$

Figure 4b,c show the results of equivalent impedance, dielectric constant, and magnetic permeability. The equivalent permittivity and permeability are close to each other, while the real part of the equivalent impedance approaches 1 and the imaginary part approaches 0. This indicates that the structure achieves excellent impedance matching with free space, enabling efficient coupling of electromagnetic wave energy into the structure to achieve broadband radar stealth performance. Simultaneously, the real part of the equivalent permittivity and permeability remain positive across the wideband spectrum, indicating that the designed absorber possesses strong attenuation capabilities. The power loss distribution shown in Figure 4d reveals that electromagnetic energy dissipation is predominantly concentrated in the resistive ITO radar absorption layer, which accounts for the major proportion of the total power dissipation. In contrast, the infrared stealth layer and PDMS dielectric layers exhibit minimal loss. This confirms that the infrared stealth layer functions as an efficient microwave transmission and impedance-modulation layer while preserving infrared suppression capability. The energy dissipation characteristic demonstrates that functional decoupling between infrared suppression and microwave absorption is successfully achieved through structural and resistive co-design.

**Figure 5:**
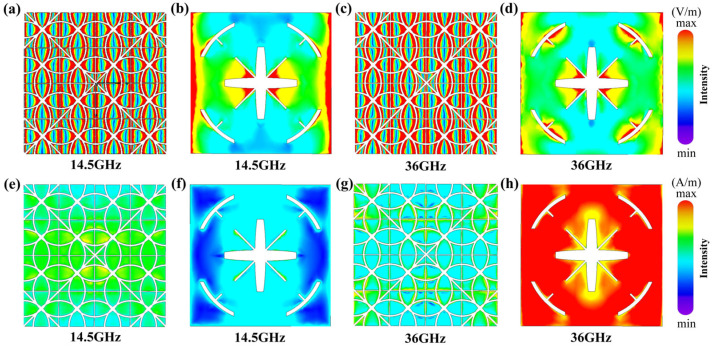
Surface electric field and magnetic field distribution of the absorber: electric field distribution at 14.5 GHz (**a**,**b**) and 36 GHz (**c**,**d**); magnetic field distribution at 14.5 GHz (**e**,**f**) and 36 GHz (**g**,**h**).

To further elucidate the broadband absorption mechanism, the distribution of electric field, magnetic field, surface current, and power loss density was analyzed at two representative absorption peak frequencies, 14.5 GHz and 36 GHz. Figure 5a–d show the electric field distributions at 14.5 GHz and 36 GHz. It can be observed that at both absorption peaks, the strong electric fields are primarily concentrated at the edges of the ITO patterns and the unit gaps within the infrared stealth layer, exhibiting distinct features of electric field localization enhancement. This indicates that the structure excites significant electrical resonance modes at both frequencies, facilitating the trapping and concentration of electromagnetic energy. For the radar absorption layer, the electric field distribution at 14.5 GHz is primarily concentrated at the gaps of the cross and the unit. While at 36 GHz, the electric field distribution is simultaneously concentrated at the rings in addition to the cross gaps. The distribution of magnetic field is shown in Figure 5e–h. The magnetic field strength is significantly stronger at 36 GHz than at 14.5 GHz and is primarily concentrated near the radar absorption layer, indicating that magnetic resonance effects dominate the absorption process at higher frequencies.

**Figure 6:**
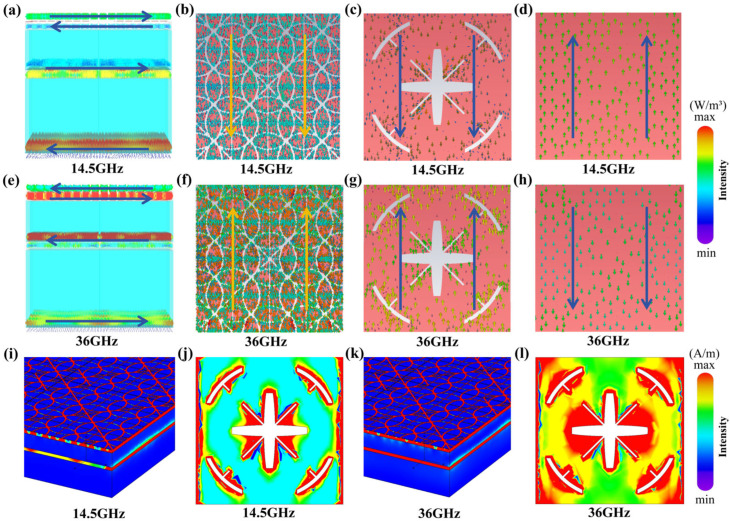
Current and power density loss distribution: side view (**a**) and top view (**b**–**d**) of current distribution at 14.5 GHz; side view (**e**) and top view (**f**–**h**) of current distribution at 36 GHz; (**i**,**j**) power density loss distribution at 14.5 GHz and (**k**,**l**) at 36 GHz. The arrows depict the overall direction of the surface current.

Figure 6a–d show the surface current distribution of the absorber. The figure shows that at a frequency of 14.5 GHz, the infrared stealth layer and radar absorption layer exhibit identical current directions. Under the same surface current direction, electrical resonance occurs between them. Meanwhile, the radar absorption layer and reflective layer generate magnetic resonance with opposite current directions. This indicates that both electric resonance and magnetic resonance coexist within the structure at the 14.5 GHz band. As shown in Figure 6e–h, at the 36 GHz frequency, the surface current directions of the radar absorption layer and the upper/lower structural layers exhibit a distinct opposite distribution, indicating that the magnetic resonance effect is more pronounced at this frequency band.

Figure 6i–l show the power loss density distribution at corresponding frequencies. It can be observed that power loss is primarily concentrated in the ITO patterned regions of the infrared stealth layer and radar absorption layer, highly overlapping with areas of high electric field intensity. This indicates that electromagnetic energy is efficiently dissipated through the ohmic loss mechanism within the resistive ITO film. The broadband absorption of the absorber simultaneously relies on the synergistic effects of electromagnetic resonance. At low frequencies, the system is dominated by electric dipole resonance, efficiently capturing energy through electric coupling. At high frequencies, the system switches to magnetic dipole resonance dominance, achieving energy convergence via magnetic coupling and induction. The coupled resonance mechanism excites strong surface currents, which induce significant ohmic losses. This synergistic process efficiently converts the gathered electromagnetic energy into thermal energy, enabling broadband, high-absorption performance even under ultra-thin constraints.

### 3.4. Polarization, Angle of Incidence, and Bending Performance

In practical applications, electromagnetic waves often strike target structures with different polarization and incident angles. Simultaneously, flexible stealth metasurfaces inevitably undergo bending deformation when applied to the equipment. Therefore, evaluating the electromagnetic performance stability of the designed radar-infrared compatible stealth metasurface under different polarization states, incidence conditions, and bending configurations is crucial for verifying its engineering applicability.

**Figure 7:**
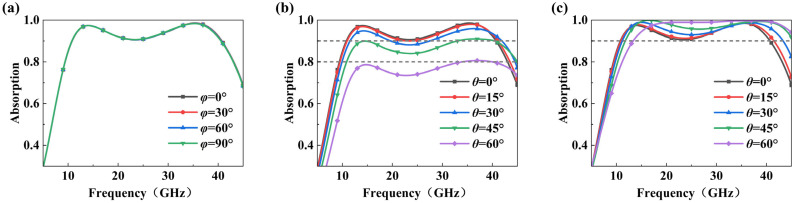
Absorption curves of the absorber: (**a**) at different polarization angles (*φ*); (**b**) at different incident angles (*θ*) in TE mode; (**c**) at different incident angles (*θ*) in TM mode.

Figure 7a shows the absorption rate-versus-frequency curve of the absorber when TE normal waves have different polarization angles (*φ*). It can be observed that the absorption remains essentially unchanged as the polarization angle increases, demonstrating the excellent polarization insensitivity of the absorber. This is primarily attributed to the absorber’s quadruple rotational symmetry design, enabling it to excite similar electromagnetic resonance modes under different polarization directions. This maintains stable impedance matching conditions and energy dissipation.

Figure 7b and c show the absorption rate variations under TE and TM polarization at incident angles of 0°, 15°, 30°, 45°, and 60°, respectively. The results indicate that for the TM mode, the absorption rate remains above 90% across the 0–60° incident angle range, with a blue shift in the absorption band. For the TE mode, the absorption rate remains above 90% when the incident angle is less than 15°, above 80% when it is less than 45°, and above 70% when it reaches 60°. This difference in angular stability stems from the fundamental distinction in how the electromagnetic vectors of TE and TM modes couple with the absorber. As the incident angle increases, the magnetic field vector in the TE mode generates a component perpendicular to the interface. This component exhibits reduced coupling efficiency with the vertical resonant modes, resulting in greater angular sensitivity. In contrast, the magnetic field vector of the TM mode remains parallel to the interface. The primary variation occurs in the electric field vector component perpendicular to the interface. This component more readily excites the structure’s inherent vertical electric resonance mode, resulting in coupling efficiency less affected by changes in the incident angle and exhibiting superior angle stability [41]. In summary, this structure demonstrates superior wide-angle absorption characteristics in the TM mode.

The designed absorber has a thickness of only 2.65 mm and utilizes a flexible PDMS substrate, which provides flexibility and enables it to bend and adhere to non-planar surfaces. Therefore, the performance of the absorber under bending conditions was further analyzed. To validate the absorption performance of the designed flexible transparent absorber under different conformal conditions, a transparent absorber model comprising 3 × 3 basic units was established to simulate the reduction in dual-station RCS. Starting from the central axis of the model, the cylindrical sections with radii of 60, 45, 20, and 10 mm were bent, using metal perfect electric conductors (PECs) of identical dimensions as reference controls. The RCS reduction is quantified as the difference between the PEC and absorber RCS expressed in dB. The incident angles are *θ* = 0°, *φ* = 0° under vertically polarized incidence conditions and *θ* = 0°, *φ* = 90° under horizontally polarized incidence conditions, as shown in Figure S2.

**Figure 8:**
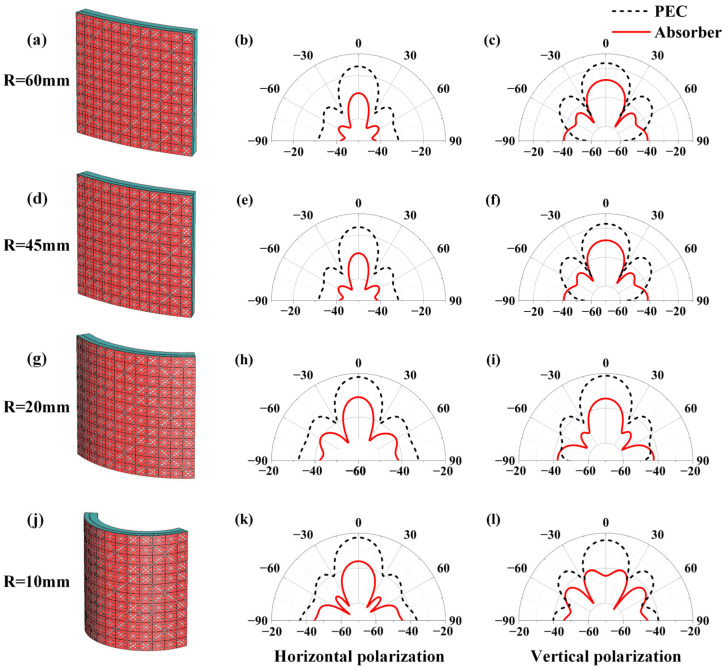
Comparison of dual-station RCS at 25 GHz between absorbers and metallic conductors under different bending states: bending radius of (**a**–**c**) 60 mm; (**d**–**f**) 45 mm; (**g**–**i**) 20 mm; (**j**–**l**) 10 mm. For each bending states, the first image (**a**,**d**,**g**,**j**) shows the structural schematic; the second (**b**,**e**,**h**,**k**) shows the polar plots of RCS under horizontal polarization; and the third (**c**,**f**,**i**,**l**) shows the polar plots of RCS under vertical polarization.

Simulation results at 25 GHz are shown in Figure 8. Compared to PEC plates, the backward RCS of transparent absorbers achieves effective reduction across a wide angular range. Its radiation pattern retains a shape like that of a PEC plate, indicating that the RCS reduction originates primarily from the absorption of incident waves rather than from scattering redistribution. As the bending angle increases, both the directivity and the peak intensity of the RCS gradually decrease. This trend is because the smaller radius of the curve effectively increases the local angle of incidence of parts of the surface, which changes the impedance matching conditions of the absorber. This mismatch modulates both the amplitude and the phase of the reflection. However, in all bending states, and regardless of polarization, the absorber maintains an omnidirectional suppression of backscattered energy relative to a PEC.

A minor exception occurs under vertical polarization at extreme oblique incidence (beyond 80°), where the scattered energy slightly exceeds that of a same-sized metal plate. This phenomenon is attributed to the grating lobes, which is an inherent feature of quasi-periodic metasurfaces at specific frequencies. A portion of the incident energy is not absorbed but instead coherently scattered into non-specular directions. The emergence of these grating lobes is independent of the unit-cell design and absorption properties, and their impact on the overall, angle-averaged RCS performance is negligible. In summary, compared to the omnidirectional RCS of a metallic perfect electric conductor, reductions exceeding 10 dB are achieved across most observation angles, demonstrating its stable performance and suitability for conformal stealth applications.

The aforementioned results show the metasurface characteristics of ultra-thinness, high mechanical flexibility, multi-frequency compatibility, and excellent environmental adaptability, as summarized in Table S1, making it a highly competitive multi-spectral stealth solution.

## 4. Conclusions

In summary, this paper designs and systematically investigates a flexible transparent radar-infrared compatible stealth metasurface. By synergistically combining a patterned ITO infrared stealth layer, a resistive radar absorption layer, and a continuous ITO reflective layer, it effectively integrates low infrared emissivity with a high broadband radar absorption performance within an ultra-thin transparent system. The results demonstrate that with a total optimized thickness of only 2.65 mm, it achieves more than 90% microwave absorption across the frequencies of 10.8 to 40.8 GHz, with a relative bandwidth of 116.3%. It covers multiple typical radar operating bands, including X, Ku, K, and Ka, while maintaining a total infrared emissivity of approximately 0.33. Furthermore, this metasurface exhibits an insensitivity to electromagnetic wave polarization and maintains a stable absorption performance within 60 degrees of incidence. It achieves significant backscatter suppression even under bending conditions, decreasing RCS by more than 10 dB over a wide angular range. These results demonstrate that the proposed flexible transparent metasurface combines ultra-thinness, mechanical flexibility, multi-frequency compatibility, and excellent environmental adaptability, offering a viable new approach for the engineering application of multi-spectral stealth and conformal electromagnetic camouflage structures.

## Figures and Tables

**Table 1:** The Metasurface Absorber Parameters.

Parameter	Value (mm)	Parameter	Value (mm)
P	8	b	0.3
t	0.175	c	3
u	0.95	e	2
n	0.1	m	1
q	0.2	w	0.1
a	2	l	7.8

## Data Availability

The data that support the findings of this study are available from the corresponding authors upon reasonable request.

## Supplementary Materials

The following supporting information can be downloaded at https://www.mdpi.com/article/10.3390/mi17030277/s1: Figure S1: Optimized design of infrared stealth layers: (a–e) unit-cell geometries with varying r; (f) corresponding ITO duty cycles; Figure S2: Schematic of the absorber in a bent state: (a) schematic of the absorber bending; (b) schematic of the Theta and phi angles; Figure S3: Design evolution of the infrared stealth unit cell and comparison of microwave transmission performance: (a) model geometries; (b) microwave transmission spectra (S21) of Models; (c) normalized transmission comparison; Figure S4: Radar absorber unit cell design: (a) stepwise structural evolution of the radar absorber unit cell, Model 1 (thick cross), Model 2 (cross with diagonal arms), Model 3 (final configuration with outer circular ring); (b) reflection coefficients (S11) corresponding to the three models; (c) absorption rates for the three models; Table S1: Comparison of the present studies with previous research [5,18,27,42,43,44].
