# Supplementary material for: Numerical Analysis and Design of an Ultra-Thin Flexible Transparent Metasurface for Broadband Radar-Infrared Compatible Stealth
Source: Micromachines (Basel). 2026 Feb 24;17(3):277. doi: 10.3390/mi17030277 (PMC13027669; doi:10.3390/mi17030277)
Supplement: Supplementary file 1 [file micromachines-17-00277-s001.zip › micromachines-4135483-supplementary.pdf]

Supporting Information

Numerical Analysis and Design of an Ultra-Thin Flexible Transparent Metasurface for Broadband Radar-Infrared Compatible Stealth

Liang Xu[a], Yijia Li[b], Xingyuan Wang[a], Jingxuan Sun[a] and Zhixun Yang[a*]*

[a] College of Mechanical and Electrical Engineering, Harbin Engineering University, Harbin 150001, People's Republic of China.

[b] College of Information and Communication Engineering, Harbin Engineering University, Harbin 150001, People's Republic of China.

*Corresponding author: Liang Xu, Zhixun Yang

E-mail: xuliang61@hrbeu.edu.cn, yangzhixun@hrbeu.edu.cn

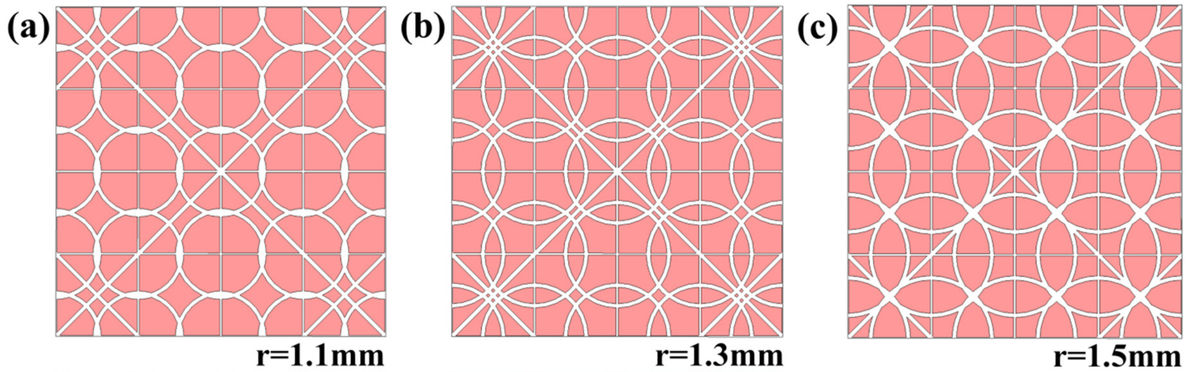

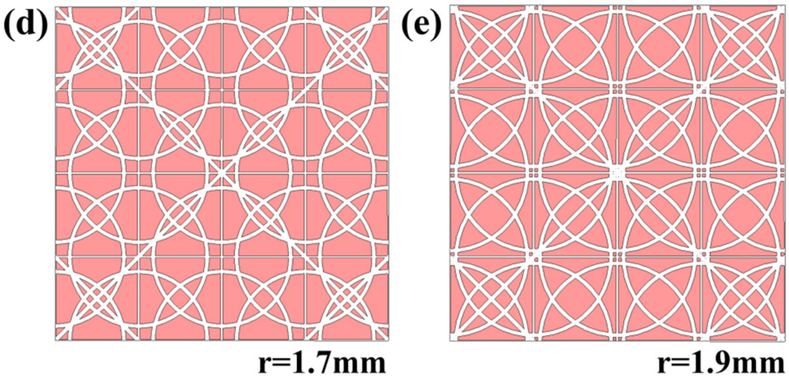

(f)

r(mm)	Duty Cycle
1.1	0.73
1.3	0.69
1.5	0.70
1.7	0.65
1.9	0.61

Figure S1. Optimized design of infrared stealth layers: (a)-(e) unit-cell geometries with varying r; (f) corresponding ITO duty cycles.

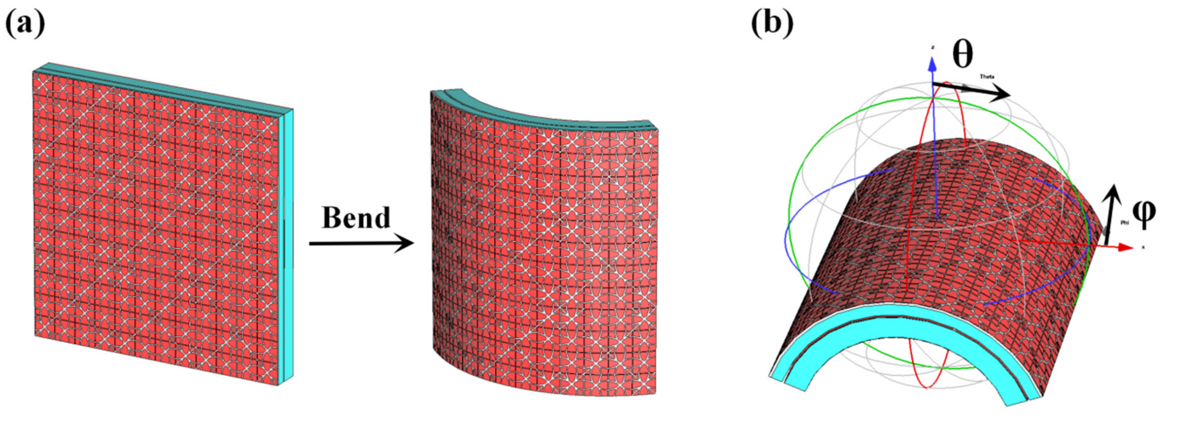

Figure S2. Schematic of the absorber in a bent state: (a) schematic of the absorber bending; (b) schematic of the theta and phi angles.

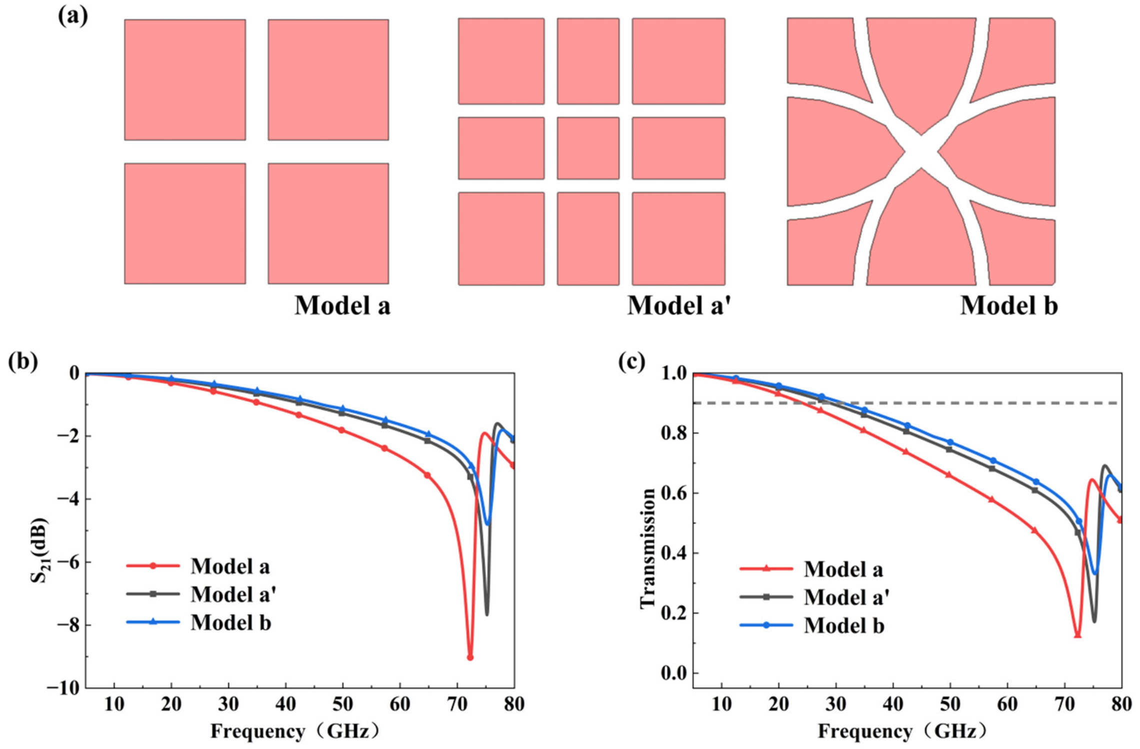

Figure S3. Design evolution of the infrared stealth unit cell and comparison of microwave transmission performance (a) model geometries; (b) microwave transmission spectra (S21) of Models; (c) normalized transmission comparison.

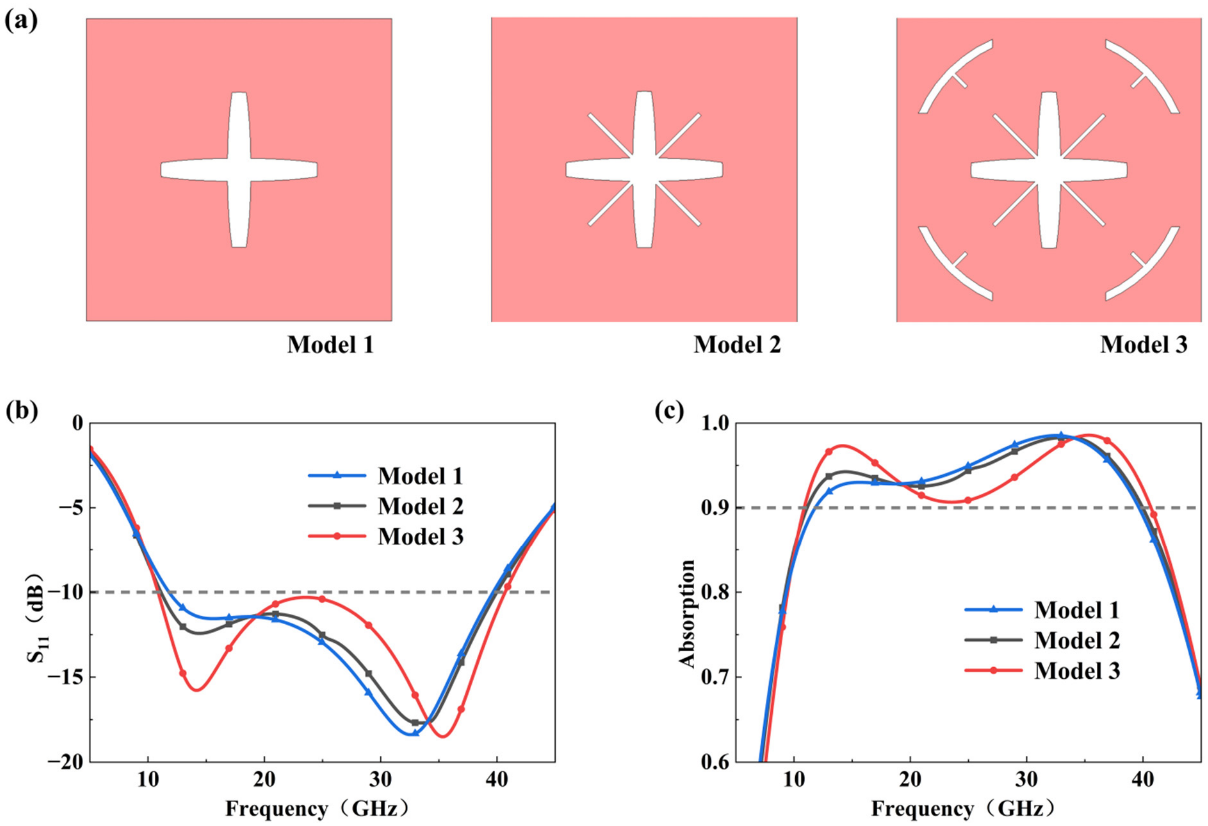

Figure S4. Radar absorber unit cell design: (a) stepwise structural evolution of the radar absorber unit cell, Model 1 (thick cross), Model 2 (cross with diagonal arms), Model 3 (final configuration with outer circular ring); (b) reflection coefficients (S11) corresponding to the three models; (c) absorption rates for the three models.

Table S1. Comparison of the present studies with previous research

Reference	Absorption Bandwidth (GHz)	Thickness (mm)	Optical Transmittance	Flexibility
[5]	7.2–26.6 (19.4)	3.525	Yes	No
[18]	7.3–18.8 (11.5)	3.5	Yes	Yes
[27]	7.6–16.4 (8.8)	3.135	Yes	No
[42]	8.7–32 (23.3)	3.5	Yes	Yes
[43]	8–18 (10.0)	2.4	Yes	No
[44]	8.6–16.3 (7.7)	4.4	No	Yes
This work	10.8–40.8 (30.0)	2.65	Yes	Yes
